# Polymorphism Located between *CPT1B* and *CHKB*, and HLA-DRB1*1501-DQB1*0602 Haplotype Confer Susceptibility to CNS Hypersomnias (Essential Hypersomnia)

**DOI:** 10.1371/journal.pone.0005394

**Published:** 2009-04-30

**Authors:** Taku Miyagawa, Makoto Honda, Minae Kawashima, Mihoko Shimada, Susumu Tanaka, Yutaka Honda, Katsushi Tokunaga

**Affiliations:** 1 Department of Human Genetics, Graduate School of Medicine, The University of Tokyo, Tokyo, Japan; 2 The Sleep Disorders Research Project, Tokyo Institute of Psychiatry, Tokyo, Japan; 3 Japan Somnology Center, Neuropsychiatric Research Institute, Tokyo, Japan; 4 Center for Narcolepsy, Stanford University School of Medicine, Palo Alto, California, United States of America; University of Cambridge, United Kingdom

## Abstract

**Background:**

SNP rs5770917 located between *CPT1B* and *CHKB*, and HLA-DRB1*1501-DQB1*0602 haplotype were previously identified as susceptibility loci for narcolepsy with cataplexy. This study was conducted in order to investigate whether these genetic markers are associated with Japanese CNS hypersomnias (essential hypersomnia: EHS) other than narcolepsy with cataplexy.

**Principal Findings:**

EHS was significantly associated with SNP rs5770917 (*P_allele_* = 3.6×10^−3^; OR = 1.56; 95% c.i.: 1.12–2.15) and HLA-DRB1*1501-DQB1*0602 haplotype (*P*_positivity_ = 9.2×10^−11^; OR = 3.97; 95% c.i.: 2.55–6.19). No interaction between the two markers (SNP rs5770917 and HLA-DRB1*1501-DQB1*0602 haplotype) was observed in EHS.

**Conclusion:**

CPT1B, CHKB and HLA are candidates for susceptibility to CNS hypersomnias (EHS), as well as narcolepsy with cataplexy.

## Introduction

We previously reported that SNP rs5770917 located between the carnitine palmitoyltransferase 1B (*CPT1B*) and choline kinase beta (*CHKB*) genes was associated with susceptibility to narcolepsy with cataplexy after performing a genome-wide association study in Japanese and Korean populations (*P* = 1.4×10^−7^; odds ratio = 1.68) [1]. In addition, significantly lower levels of both *CPT1B* and *CHKB* mRNA expression were observed in heterozygotes (TC) with the risk allele, as compared to homozygotes (TT) with the major allele [1]. Moreover, it is noteworthy that all narcoleptic patients with cataplexy in Japan carry a human leukocyte antigen (HLA)-DRB1*1501-DQB1*0602 haplotype [2], [3]. Similar findings have been confirmed in individuals of European and African decent for whom the association is with DQB1*0602. [3], [4].

Central nervous system (CNS) hypersomnias other than narcolepsy with cataplexy are also complex disorders. Both genetic and environmental factors may contribute to the development of CNS hypersomnias, as in the case of narcolepsy with cataplexy [5], [6]. Identification of the genetic factors has been challenging because of the low prevalence and the difficulty of diagnosis [5], [6]. Although several reports have confirmed the association between some CNS hypersomnias and HLA, HLA typing was performed at the low-resolution serological level, and the association showed marginally statistical significance (0.01<*P*<0.05) because the number of cases was small [6]–[9]. Thus, in this study, we extended previous association studies to essential hypersomnia (EHS), which is a group of other CNS hypersomnias similar to narcolepsy with cataplexy in the symptom of excessive daytime sleepiness. Our diagnostic criteria for EHS comprised three clinical items: 1) recurrent daytime sleep episodes that occur basically every day over a period of at least 6 months; 2) absence of cataplexy; 3) the condition does not meet the diagnostic criteria of any other disorder causing excessive daytime sleepiness, such as sleep-apnea syndrome [9], [10]. EHS is heterogeneous and consists of several CNS hypersomnias from aborted form of narcolepsy with cataplexy, narcolepsy without cataplexy and a part of idiopathic hypersomnia without long sleep time if we employed International Classification of Sleep Disorders 2^nd^ edition criteria (AASM 2005). Our diagnostic criteria for EHS are focused on the daytime clinical characteristics of CNS hypersomnias [9], [10]. A case-control association study was conducted in order to examine whether SNP rs5770917 and HLA-DRB1*1501-DQB1*0602 haplotype are associated with EHS.

## Methods

Cases of EHS (n = 137) and Controls (n = 569) were unrelated Japanese living in Tokyo or neighboring areas. Written informed consent was obtained from participants, and the study was approved by the local institutional review boards of all collaborative organizations. Our diagnostic criteria for EHS comprised three clinical items: 1) recurrent daytime sleep episodes that occur basically every day over a period of at least 6 months; 2) absence of cataplexy; 3) the condition does not meet the diagnostic criteria of any other disorder causing excessive daytime sleepiness, such as sleep-apnea syndrome [9], [10].

Genotyping for SNP rs5770917 in cases was performed using Taqman genotyping assays. For controls, we used the genotype data from the previous genome-wide association study for narcolepsy with cataplexy [1]. Genotyping for *HLA-DRB1* and *HLA-DQB1* in cases was performed by Luminex Multi-Analyte Profiling system (xMAP) with a WAKFlow HLA typing kit (Wakunaga, Hiroshima, Japan). Briefly, target DNA was amplified by polymerase chain reaction (PCR) with biotinylated primers. The PCR amplicon was then denatured and hybridized to complementary oligonucleotide probes immobilized on fluorescent coded microsphere beads. At the same time, biotinylated PCR products were labeled with phycoerythrin-conjugated streptavidin and immediately examined with Luminex 100 (Luminex, Austin, TX). We did not perform HLA-typing in controls, instead we utilized *HLA-DRB1* and *HLA-DQB1* frequency data obtained from Japanese Society for Histocompatibility and Immunogenetics databank (a total of 516 Japanese general population were genotyped) (http://jshi.umin.ac.jp/mhc/mhc_vol06-10/v08nakajima_all.pdf).

The observed associations of SNP rs5770917 and HLA-DRB1*1501-DQB1*0602 haplotype were assessed by comparing frequency differences between cases and controls using chi-squared test. Population attributable risk percentage (PAR) for the risk genotypes (SNP rs5770917 C/C and T/C) was evaluated using the following formula, PAR = [p(RR–1)]/[p(RR–1)+1]), where p is calculated based on genotype frequencies in healthy controls and RR indicates relative risk of the risk genotypes. Odds ratio (OR) of the risk genotypes is similar to RR because of the low prevalence of EHS and narcolepsy with cataplexy. Interactions between the two markers (SNP rs5770917 and HLA-DRB1*1501-DQB1*0602 haplotype) were calculated based on the positivity of each risk allele in EHS using the correlation coefficient.

## Results

In order to investigate whether SNP rs5770917 is also a genetic risk marker for the development of EHS, the SNP was genotyped in 137 cases and 569 controls. We found a significant difference between cases and controls (*P_allele_* = 3.6×10^−3^; OR = 1.56; 95% c.i.: 1.12–2.15) (Table 1). Significant deviation from Hardy-Weinberg equilibrium (HWE) was not observed in either cases or controls. To estimate the epidemiological significance of SNP rs5770917 for EHS development in the Japanese population, PAR was found to be 14.2% in our Japanese samples (Table 2).

**Table 1:** Frequency of SNP rs5770917 in Japanese cases and controls.

	SNP rs5770917			HWE *P*	RAF	OR (95% c.i.)	*P_allele_*
	CC (%)	TC (%)	TT (%)				
Case	8 (5.8%)	46 (33.6%)	83 (60.6%)	0.631	22.6%	1.56 (1.12–2.15)	3.6×10^−3^
Control	13 (2.3%)	154 (27.1%)	402 (70.7%)	0.697	15.8%		

*P*_allele_ was calculated by comparing allele frequency differences in SNP rs5770917 between cases and controls using chi-squared test (one-tailed test).

RAF, risk allele frequency (C allele); OR, odds ratio; 95% c.i., confidence interval.

HWE *P* represents P value from chi-squared test for Hardy-Weinberg equilibrium.

**Table 2:** Population attributable risk in SNP rs5770917.

	SNP rs5770917		OR (95% c.i.)	PAR
	C+ (%)	C− (%)		
EHS	54 (39.4%)	83 (60.6%)	1.57(1.06–2.31)	14.2%
Narcolepsy with cataplexy	169 (44.4%)	212 (55.6%)	1.92(1.46–2.52)	21.2%

EHS, essential hypersomnia; OR, odds ratio; 95% c.i., confidence interval; PAR, population attributable risk.

We also conducted an association study for HLA-DRB1*1501-DQB1*0602 haplotype. Cases carried a significantly higher frequency of HLA-DRB1*1501-DQB1*0602 haplotype than the Japanese general population (*P*_positivity_ = 9.2×10^−11^; OR = 3.97; 95% c.i.: 2.55–6.19) (Table 3).

**Table 3:** Positivity for HLA-DRB1*1501-DQB1*0602 haplotype.

	HLA-DRB1*1501-DQB1*0602 haplotype		OR (95% c.i.)	*P*_positivity_
	+ (%)	− (%)		
Case	47 (34.3%)	90 (65.7%)	3.97 (2.55–6.19)	9.2×10^−11^
Control	60 (11.6%)	456 (88.4%)		

*P*_positivity_ was calculated by comparing the differnce in positivity for HLA-DRB1*1501-DQB1*0602 haplotype between cases and controls using chi-squared test (one-tailed test).

OR, odds ratio; 95% c.i., confidence interval.

The interaction between these two markers (SNP rs5770917 and HLA-DRB1*1501-DQB1*0602 haplotype) was then calculated in EHS. No significant interaction was found (*P* = 0.20; *r^2^* = 0.01), thus suggesting that these markers independently affect susceptibility to EHS.

## Discussion

In the present study, SNP rs5770917, which was identified in our previous genome-wide association study of narcolepsy with cataplexy [1], was also found to confer susceptibility to EHS, a group of other CNS hypersomnias similar to narcolepsy with cataplexy. Thus, the SNP is considered to be a common risk marker for CNS hypersomnias in general. Nevertheless, the OR for narcolepsy with cataplexy (1.92) was greater than that for EHS (1.57) (Table 2). Moreover, PAR for narcolepsy with cataplexy (21.2%) was higher than that for EHS (14.2%) (Table 2), because the risk allele frequency of the SNP in narcolepsy with cataplexy (25.2%) was greater than that in EHS (22.6%) (Table 1) [1].

CPT1B is the rate-controlling enzyme of long-chain fatty acid β-oxidation in muscle mitochondria. CPT1B catalyzes the transport of long-chain fatty acyl-CoAs from the cytoplasm into the mitochondria through the carnitine shuttle [11]. Several reports have indicated the role of fatty acid β-oxidation and carnitine system in sleep regulation. First, fasted juvenile visceral steatosis (*jvs* ^−/−^) mice with systemic carnitine deficiency exhibit a higher frequency of fragmented wakefulness and rapid eye movement (REM) sleep, and reduced locomotor activity [12], [13]. These phenotypes in fasted *jvs* ^−/−^ mice are similar to those in mouse models of narcolepsy [14], [15]. Moreover, fasted *jvs^−/−^* mice are activated by modafinil, which is used for the treatment of human narcolepsy [12]. Second, mice deficient in short-chain acyl-CoA dehydrogenase (encoded by Acads), an enzyme catalyzing the first step of β-oxidation, have shown significantly slower theta frequency during REM sleep [16]. Administration of acetyl-L-carnitine, which is known to restore β-oxidation in the mitochondria, significantly recovers slow theta frequency in mutant mice [16].

CHKB catalyzes the first phosphorylation reaction in the CDP-choline pathway for phosphatidylcholine synthesis [17]. Phosphatidylcholine is the major membrane phospholipid in mammalian cells [18] and inhibition of phosphatidylcholine synthesis triggers apoptosis in the brain [19]. CDP-choline, which is used in the treatment of disorders of a cerebrovascular nature [20], increases acetylcholine release [21]. Acetylcholine is a known REM- and wake-promoting neurotransmitter that increases narcolepsy symptoms [22]. Thus, either of these two genes (*CPT1B* and *CHKB*) is a plausible candidate for susceptibility to CNS hypersomnias (EHS), as well as narcolepsy with cataplexy.

To date, reports on the association between HLA and CNS hypersomnias other than narcolepsy with cataplexy have been based on serological typing and small sample size, thus yielding only marginal statistical significance [6]–[9]. In this study, we found a highly significant association between HLA and EHS using DNA-based high-resolution typing (*P*_positivity_ = 9.2×10^−11^), suggesting an immunological pathogenesis for EHS.

It is important to examine the interaction between SNP rs5770917 and HLA with regard to susceptibility to CNS hypersomnias. However, it is impossible to calculate the interaction between SNP rs5770917 and HLA in narcolepsy with cataplexy, because almost all patients possess HLA-DRB1*1501-DQB1*0602 haplotype. No interaction was observed between SNP rs5770917 and HLA-DRB1*1501-DQB1*0602 haplotype, suggesting that they are independent risk factors for not only EHS but also narcolepsy with cataplexy.

EHS analyzed in this study have recurrent daytime sleep episodes but do not have cataplexy. Thus, SNP rs5770917 located between *CPT1B* and *CHKB* might be involved in the pathogenesis of excessive daytime sleepiness, not cataplexy. Although our cases are heterogeneous, as exemplified by the findings that not all patients with EHS carry HLA-DRB1*1501-DQB1*0602 haplotype, SNP rs5770917 is considered to be a common risk marker for both HLA positive and negative EHS, because no interaction was observed between SNP rs5770917 and HLA-DRB1*1501-DQB1*0602 haplotype.

Our data suggest that EHS, a group of heterogeneous CNS hypersomnias similar to narcolepsy with cataplexy, might be in continuity with narcolepsy with cataplexy in common genetic background.
